# Atmospheric carbon dioxide concentration as fuel gauge

**DOI:** 10.1038/s41598-025-98459-1

**Published:** 2025-04-25

**Authors:** Balázs M. Fekete

**Affiliations:** 1 Department of Civil Engineering, City College of New York, City University of New York, New York, USA; 2 Environmental Sciences Initiative, Advanced Science Research Center at the Graduate Center, City University of New York, New York, USA

**Keywords:** Carbon capture and storage, Energy storage, Fossil fuels, Palaeoclimate

## Abstract

Climate change skeptics like to point to the high atmospheric carbon dioxide concentration in paleo records as “*proof*” of the exaggeration of the climate change agenda. Instead of debunking these arguments, the present paper represents a thought experiment that considers the atmospheric carbon dioxide concentration in the past as a proxy for fossil fuel reserves and interprets the contemporary rise in carbon dioxide concentration as a fuel gauge to estimate the exhaustion of the remaining fossil fuel reserves under different energy consumption scenarios. The resulting conclusion is that the dangers of exhausting the remaining fossil fuel resources are likely on par with the anticipated adverse effects of climate change and should convince even climate change skeptics that the transition to non-fossil energy sources is inevitable and urgent since the remaining fossil fuel reserves will be likely exhausted in less than a century. The presented analysis goes further than the currently adopted “*net-zero*” ambitions. It demonstrates that there is no room for compromise and the transition has to be “*true-zero*” use of fossil fuels. Accounting gimmicks like carbon capture and sequestration or carbon trading are just as unsustainable as the continued reliance on fossil fuels.

## Introduction

**Fig. 1:**
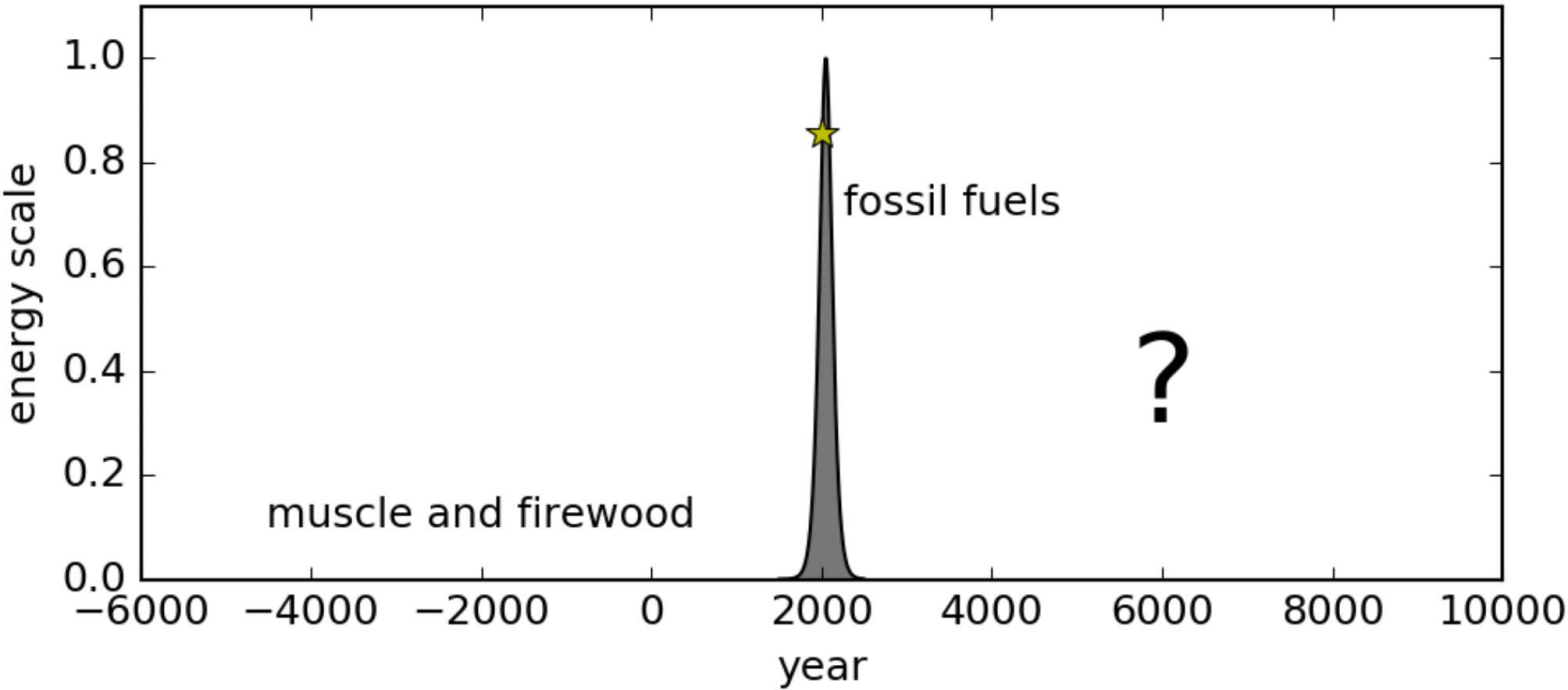
“*Schematic view of the human energy production*” from Murphy et al.^1^.

Industrial societies powered by fossil fuels are unsustainable. Murphy et al.^1^ eloquently articulated the incompatibility of modernity with planetary limits. The schematic view of human energy production (Fig. 1) depicts the alarming reality of the inevitable ending of the fossil fuel era in the not-too-distant future.

One of the arguments questioning the significance of carbon dioxide emissions^2^ is that atmospheric carbon dioxide concentration in the past far exceeded current and anticipated future levels. Patrick Moore, an ecologist who identifies as a “*Greenpeace dropout*”^3^ goes as far as to argue that plants currently suffocate and the “*liberation*” of fossilized carbon prevented our planet from turning into an iceball^3^.

Opponents of the climate change agenda often accuse activists of exaggeration and tend to dispute the severity of the consequences of climate change^4^. Even if the climate change impacts are somewhat overstated, Murphy^5^ makes an excellent argument by pointing out that the story of the “*boy who cried wolf*” is largely misunderstood. While this story is often viewed as an example of false alarms, the true moral is that the wolf comes. It is hard to see climate change remain a benign problem when the atmospheric carbon dioxide concentration is rapidly approaching its first doubling since the beginning of the industrial revolution^6^.

Instead of debunking the arguments disputing the severity of climate change, this paper presents a thought experiment if the upper limit to continued carbon dioxide emission was the atmospheric carbon dioxide concentration around the Cambrian explosion 420 million years ago. Consequently, the paper assumes that the difference in the atmospheric carbon dioxide content during the Cambrian ($\:{C}_{paleo}=2000\left[ppm\right]$)^7^ vs. the pre-industrial era ($\:{C}_{base}=278\left[ppm\right]$) was proportional to the fossil fuel reserves, and the rise to the present level ($\:{C}_{pres}=421\left[ppm\right]$) can be interpreted as a fuel gauge showing the exhausted fossil fuel resources. While this interpretation may seem overly simplistic but appears to be consistent with the current understanding of the temporal evolution of the atmospheric carbon dioxide concentration and fossil fuel consumption. Our assumption offers a crude means to put a temporal bound to Fig. 1 and estimate the plausible pathways to exhaust the remaining reserves.

The analysis presented here was initially meant for educational purposes in the context of teaching a course on Energy and Environment at the City College of New York, and it was intentionally kept as simple as possible to allow its replication even in a classroom setting. The paper was inspired by two excellent books (by D. MacKay^8^ and T. Murphy^5^) explaining complex energy questions in a series of easy-to-follow “*back-of-the-envelop*” calculations. Reducing the discussion of atmospheric carbon dioxide concentration and energy consumption into simple equations with straightforward analytic solutions offers much-needed clarity to the scientific communication regarding climate change and our energy future.

## Results

The Brundtland Report^9^ defines sustainable development as a pathway that “*meets the needs of the present without compromising the ability of future generations to meet their own needs*”. While this definition might imply eternity, a realistic compromise might be an aim for a future that can last at least as long as the human civilization in the past (i.e. several thousand years). The first-order objective of the paper was to assess the life span of our industrial societies (Fig. 1) by linking the difference between atmospheric carbon dioxide concentration in geological times (specifically the Cambrian Explosion when terrestrial plants evolved) and pre-industrial era (as a plausible limit for fossil fuels that ever existed) in contrast to the present as a fuel gauge indicating the exhausted fossil fuel.

In addition, to considering fossil fuel resources, the paper attempts to clarify the carbon dioxide emission scenarios expressed as “*representative concentration pathways*” and corresponding “*shared socioeconomic pathways*” by relating fossil fuel consumption to carbon dioxide emission and the exhaustion of the fossil fuel reserves.

### Contrasting atmospheric carbon dioxide concentration and fossil fuel reserves

Foster et al.^7^ assembled probably the most comprehensive reconstruction of the atmospheric carbon dioxide concentration for the past 420 million years, resulting from the combination of abiotic (carbonate-silicate cycle) weathering and carbonate biomineralization. They found remarkable correspondence between the increase in solar luminosity ($\:\varDelta\:{F}_{sol}\cong\:9\left[W{\:m}^{-2}\right]$) during this 420 million years period and a decline in radiative forcing ($\:\varDelta\:{F}_{{CO}_{2}}\sim-10\left[W{\:m}^{-2}\right]$) arising from the decreasing atmospheric carbon dioxide concentration from paleo records ($\:{\:C}_{paleo}=2000\left[ppm\right]$) to the pre-industrial ($\:{C}_{base}=278\left[ppm\right]$) level. This atmospheric carbon dioxide concentration difference between geological time scales ($\:{C}_{paleo}$) and before the Industrial Revolution ($\:{C}_{base}$) appears to offer a crude estimate of the fossil fuel reserves ($\:\varDelta\:{C}_{paleo}={\:C}_{paleo}-{\:C}_{base}=1773\left[ppm\right]$) corresponding to $\:{\varDelta\:m}_{paleo{,CO}_{2}}=\text{13,466}\left[Gt\right]$ carbon dioxide containing $\:{\varDelta\:m}_{paleo,\text{C}}=3672\left[Gt\right]$ carbon (Sect. 4.1).

According to Murphy^5^, the proven coal, oil, and gas reserves are 20[ZJ] (634[TWyr]), 10[ZJ] (317[TWyr]), and 8[ZJ] (254[TWyr]) respectively totaling to 38[ZJ] (1204[TWyr]) that would lead to 2236[Gt], 643[Gt], and 396[Gt] carbon dioxide emissions when fully exhausted considering their respective carbon intensities (Sect. 4.1) totaling to 3275[Gt] carbon dioxide containing 893[Gt] carbon. A recent study estimated^10^ the ultimately recoverable coal, oil and gas resources as 14.5–31.5[ZJ] (460–999[TWyr]), 20.0–43.2[ZJ] (634–1370[TWyr]) and 13.9–46.7[ZJ] (441–1481[TWyr]) respectively totaling to 48–121[ZJ] (1535–3850[TWyr]) energy. These recoverable resources correspond to 1621–3521[Gt] 1286–2777[Gt] and 688–2313[Gt] carbon dioxide emissions by the three fuel types totaling 3595–8611[Gt] carbon dioxide with 980–2348[Gt] carbon content. In contrast, IPCC anticipates cumulative coal production up by 2100 as high as 3500[Gt]. The atmospheric carbon dioxide concentration difference between paleo^7^ records ($\:{C}_{paleo}=2000\left[ppm\right]$) and the present time ($\:{\:C}_{pres}=421\left[ppm\right]$) corresponds to $\:{\varDelta\:m}_{{res,CO}_{2}}=\text{12,348}\left[Gt\right]$ total carbon dioxide amount (Sect. 4.1) with $\:{\varDelta\:m}_{res,C}=3368\left[Gt\right]$ carbon content that appears to lead to a high approximation for the remaining fossil fuels.

**Table 1:** Energy consumption projections for the IPCC representative pathways scenarios based on atmospheric carbon dioxide concentration and different consumption trajectories.

			Unit	Present	RCP 2.6	RCP 4.5	RCP 7.0	RCP 8.5	Paleo
		Temperature increase—radiative	℃	0.6	0.7	1.2	1.9	2.2	3.2
		Temperature increase—climate	℃	1.8	2.1	3.6	5.6	6.8	9.6
		Carbon dioxide concentration	ppm	421	446	618	931	1179	2000
		Carbon increment	Gt	305	358	725	1392	1921	3673
		Energy consumption at peak	TW	21	25	50	96	132	253
		Exhausted energy at peak	TWyr	885	1039	2105	4042	5577	10,663
		Consumed energy at peak	%	8.3%	9.7%	19.7%	37.9%	52.3%	100.0%
		Remaining energy after peak	%	91.7%	90.3%	80.3%	62.1%	47.7%	0.0%
		Constant consumption to 2100	TW	0	2	16	42	63	130
		Growth years	yrs	0	7	37	64	78	105
		Constant years	yrs	466	391	171	69	38	0
		Carbon capture and sequestration	Gt yr^−1^	27	31	63	121	167	0
		Years left	**yrs**	**466**	**397**	**208**	**133**	**116**	**105**
EROI Decline	Slow	Energy cost	%	4.2%	4.9%	9.9%	19.0%	26.2%	50.0%
		Net energy consumption	TW	20	23	45	78	98	126
		Constant years	yrs	354	298	133	56	32	0
		Years left	**yrs**	**354**	**305**	**170**	**120**	**110**	**105**
	Medium	Energy cost	%	8.3%	9.7%	19.7%	37.9%	52.3%	100%
		Net energy consumption	TW	19	22	40	60	63	0
		Constant years	yrs	233	195	86	35	19	0
		Years left	**yrs**	**233**	**202**	**122**	**99**	**97**	**105**
	Rapid	Energy cost	%	16.6%	19.5%	39.5%	75.8%	104.6%	200.0%
		Net energy consumption	TW	18	20	30	23	0	0
		Constant years	yrs	106	87	32	7	-2	-29
		Years left	**yrs**	**106**	**94**	**69**	**71**	**76**	**76**

The rise of carbon dioxide concentration ($\:\varDelta\:{C}_{pres}={\:C}_{pres}-{\:C}_{base}$) from pre-industrial to the present ($\:{C}_{pres}=421\left[ppm\right]$) represents $\:{\varDelta\:m}_{pres{,CO}_{2}\:}=1118\left[Gt\right]$ added carbon dioxide with $\:{\varDelta\:m}_{pres,C\:}=305\left[Gt\right]$ carbon content. According to the Global Carbon Budget, this added carbon dioxide represents 44% of total carbon dioxide emissions ($\:{\varDelta\:m}_{{tot,CO}_{2}}=2541.5\left[Gt\right]$) between 1850 and 2019, taken up by the atmosphere, oceans, and terrestrial ecosystems. One-third of this total carbon dioxide emission was due to land-use change, and the rest $\:{\varDelta\:m}_{{fossil,CO}_{2}}=1694\left[Gt\right]$ originated from burning fossil fuels. The energy content of this emitted carbon dioxide is $\:{E}_{fossil,C}=481\left[TWyr\right]$ (Sect. 4.1). Considering the *carbon portion* of the entire energy mix ($\:{I}_{C}=56.6\%$, Sect. 4.1), the total exhausted energy derived from the carbon budget becomes $\:{E}_{pres,CB}=\frac{{E}_{fossil,C}}{{I}_{C}}=849\left[TWyr\right]$.

The narrow oscillation ($\:\varDelta\:{C}_{Hol}=120\left[ppm\right]$) of the atmospheric carbon dioxide concentration during the Holocene^11^ between 180[ppm] (during the ice ages) and 300[ppm] (in warmer periods) suggests that likely limit to biotic carbon capture and sequestration is $\:\varDelta\:{m}_{Hol,C{O}_{2}}=938\left[Gt\right]$ with $\:\varDelta\:{m}_{Hol,C}=255\left[Gt\right]$ carbon content that is tiny compared to the fossil fuel reserves.

### Exponential energy consumption trajectories

According to Murphy et al.^5^, global energy consumption follows an exponential growth at the rate of 2.4% per year corresponding to a 29-year doubling time. Conveniently, this represents a ten-fold increase per century. Considering current energy consumption ($\:{P}_{pres}=21\left[TW\right]$) at $\:r=2.4\%$ growth rate leads to $\:{E}_{pres}=\frac{{P}_{pres}}{\text{ln}\left(1+r\right)}=885\left[TWyr\right]$ energy used up to the present (Sect. 4.2). This estimate is remarkably close to $\:{E}_{pres,CB}=849\left[TWyr\right]$ derived from the carbon budget in the previous section.

One has to note that climate change deniers successfully confused the public with their assertion that the anthropogenic nature of the rising atmospheric carbon dioxide concentrations was unproven. There were some uncertainties around the turn of the 21st century when the atmospheric carbon dioxide concentration was still within its oscillation range during the Holocene and a large portion of the emitted carbon dioxide was captured biotically. During this time, scientists wrestled with the “*missing carbon sink dilemma*”^12,13^, but ultimately that was resolved. A more recent study^6^ found that when the pre-industrial atmospheric carbon dioxide concentration is deducted from the records, the remaining time series shows an accelerating rise from 1[ppm yr^− 1^] before 1970 to 2[ppm yr^− 1^] at present that corresponds to 2% per year leading to an approximately 30 years doubling time. This is remarkably similar to the exponential growth in energy consumption.

### Exhaustion of fossil fuels from atmospheric carbon dioxide concentrations

Given that the increase in atmospheric carbon dioxide concentration is primarily the result of carbon dioxide accumulation since the beginning of the Industrial Revolution, it is reasonable to assume that the concentration increase from the pre-industrial ($\:{C}_{base}=278\left[ppm\right]$) to the present ($\:{C}_{pres}=421\left[ppm\right]$) is proportional to the energy consumed $\:{E}_{pres}=885\left[TWyr\right]$ during the same time. Exhausted energy can be estimated at any atmospheric carbon dioxide concentration ($\:C$), assuming a linear relationship $\:E={\frac{C-{\:C}_{base}}{{C}_{pres}-{\:C}_{base}}E}_{pres}$ yielding (Table 1: *exhausted energy at peak*). Given the nature of the exponential growth, the consumption and the accumulated energy grow at the same rate; therefore, the equation above applies to energy consumption $\:P={\frac{C-{\:C}_{base}}{{C}_{pres}-{\:C}_{base}}P}_{pres}$ (Sect. 4.3) (Table 1: *energy consumption at peak*).

The exhausted energy ($\:{E}_{2000ppm}=\text{10,663}\left[TWyr\right]$), when the carbon dioxide concentrations return to paleo levels, provides a crude estimate of the total amount of energy consumed by the time all the fossil fuels will be exhausted. Subtracting the present exhausted energy ($\:{E}_{pres}=885\left[TWyr\right]$) offers an estimate of the remaining reserves ($\:{E}_{res}=9777\left[TWyr\right]$). This is 2.5 times higher than the highest estimate of the ultimately recoverable resources (3850[TWyr]) since it assumes that proportionalities in the carbon budget through 1850–2019 remain constant into the future. Consequently, all the estimates based on carbon dioxide concentration presented in this paper are wildly optimistic.

### Radiative forcing from carbon dioxide concentration

Repeated concerns in public dialog are the perceived shortcomings of science communications. While there is some truth to the difficulties in communicating complex scientific problems, Albert Einstein once noted, “*If you can’t explain it to a six-year-old*,* you don’t understand it yourself*.” Although one would wonder how many six-year-olds understood general relativity from Einstein’s explanation, scientists undeniably bear some responsibility for obfuscated language.

**Fig. 2:**
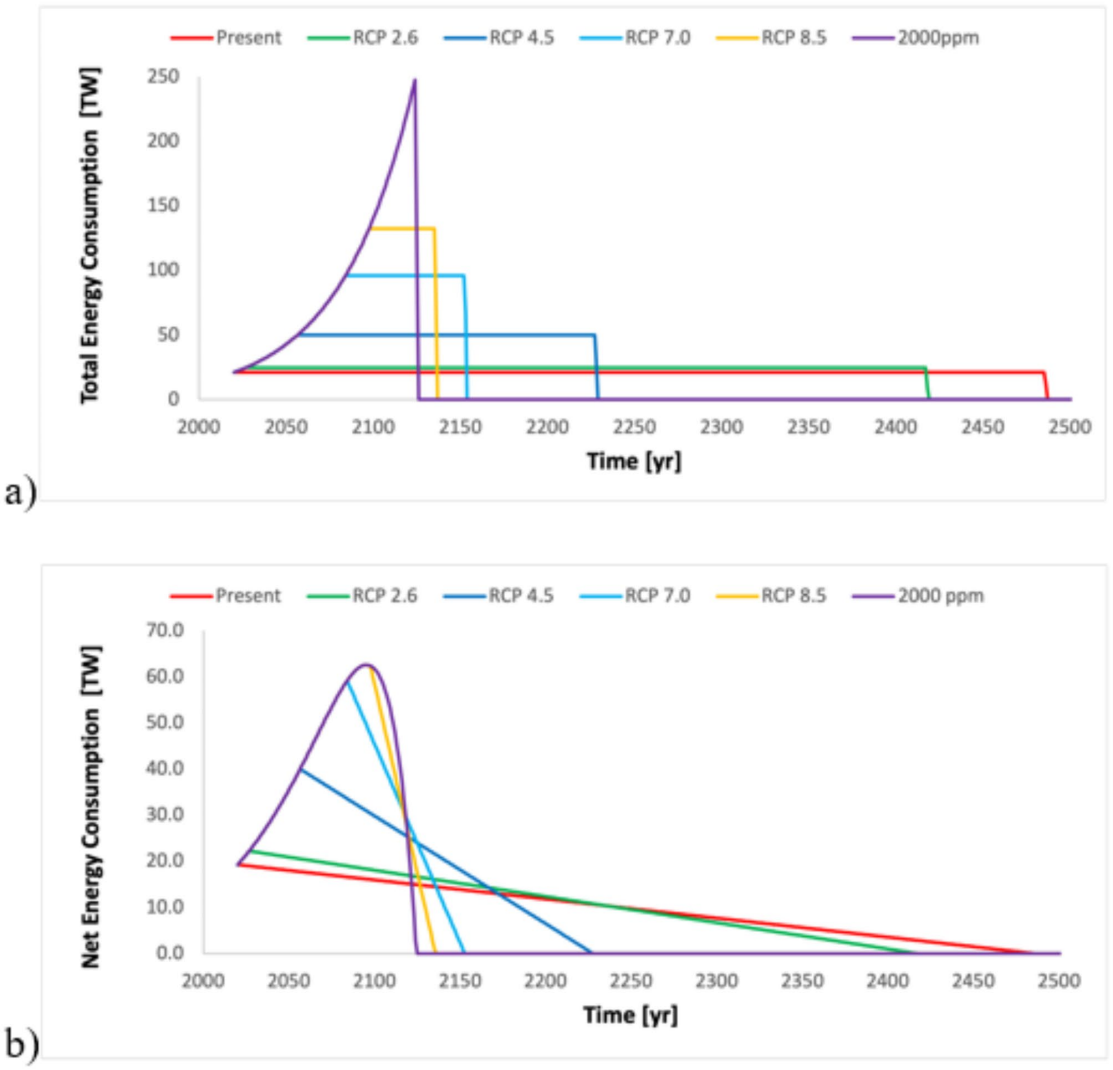
Energy consumption scenarios corresponding to representative concentration pathways. Panel (**a**) shows total energy consumption growing exponentially until the RCP limits are reached followed by constant consumption. Panel (**b**) shows the net energy consumption factoring in a medium decline in EROI, where the energy cost of energy production as a percentage of the produced energy is equal to the percentage of exhausted fossil fuel reserves.

A good example of “obfuscation” is the concept of “representative concentration pathway” (RCP) adopted in recent reports of the Intergovernmental panel on climate change (IPCC). Speaking “*acronyms*” is a questionable practice in the first place, but RCP adds further insult to injury by expressing concentration in energy imbalance^14^ ($\:\varDelta\:F=2.6,\:\:4.5,\:\:7.0,\:\:8.5\left[W{\:m}^{-2}\right]$) presumably reached by 2100 resulting from elevated atmospheric carbon dioxide concentration. These imbalances alone would lead to considerable temperature increases based on the blackbody radiation of the Earth’s surface alone (Table 1: *temperature increase—radiative*, Section 4.4), but more realistic estimates can be computed from applying $\Delta T_{{clim}} = 0.8\left[ {^\circ {\text{C}}~W^{{ - 1}} m^{2} } \right]\Delta F$ climate sensitivity parameter Table 1: *temperature increase—climate*).

A straightforward discussion of future climate scenarios would express RCPs in atmospheric carbon dioxide concentrations. Conveniently, empirical equations relate the concentration of various greenhouse gases to radiative forcing^15^ that can be inverted to express the atmospheric carbon dioxide concentrations (Sect. 4.4) corresponding to RCP scenarios (Table 1*carbon dioxide concentration*). The carbon dioxide concentrations, in turn, can be translated to added carbon content and increment since the Industrial Revolution (Table 1*carbon increment*).

One has to note that the empirical relationship relating atmospheric carbon dioxide concentration to radiative forcing combined with climate sensitivity $\left( {0.8\left[ {^\circ {\text{C}}~W^{{ - 1}} m^{2} } \right]} \right)$ leads to a 1.8 °C temperature rise at present atmospheric carbon dioxide concentration that already exceeds the 1.5 °C target of the Paris Treaty.

As an experiment, we consider the RCP scenarios as an atmospheric carbon dioxide concentration limit for exponential growth to find peak energy consumption and exhausted energy (Table 1*energy consumption at peak* and *exhausted energy at peak*). The exponential growth approximation of the energy consumption trajectories allows the computation of the time remaining before the different atmospheric carbon dioxide concentration levels are reached (Table 1: *growth years*). Notably, only reaching RCP 8.5 will take more than 75 years at the 2.4% growth rate. Assuming that the exponential growth years are followed by constant energy consumption, the remaining time for complete exhaustion of fossil fuel reserves can be computed (Table 1: *constant years*), and the sum of the growth years and the constant years provide a first-order estimate for the time left before the fossil fuel era will end (Fig. 2; Table 1: *years left*).

These simple calculations can be interpreted as storylines of continued use of fossil fuels with exponential growth until the RCP targets are reached, followed by a steady consumption until the fossil fuel reserves are exhausted (Fig. 2). The carbon dioxide concentrations do not stop in these scenarios at the different RCP-dictated levels, but they represent a turning point from exponential growth to steady energy consumption. The difference between the total energy consumed up to the different RCPs and the present allows (Table 1: *energy increments from present*) the computation of the allowable energy consumption in the next 75 years to stay within the RCPs by 2100 (Table 1: *constant consumption to 2100*). For instance, staying at RCP 2.6, which is the closest to meeting the Paris Accord, would require a tenfold immediate drop in fossil fuel consumption and sustaining that fossil fuel consumption to 2100 when it should drop further down to zero.

### Per capita energy consumption as socio-economic metric

**Fig. 3:**
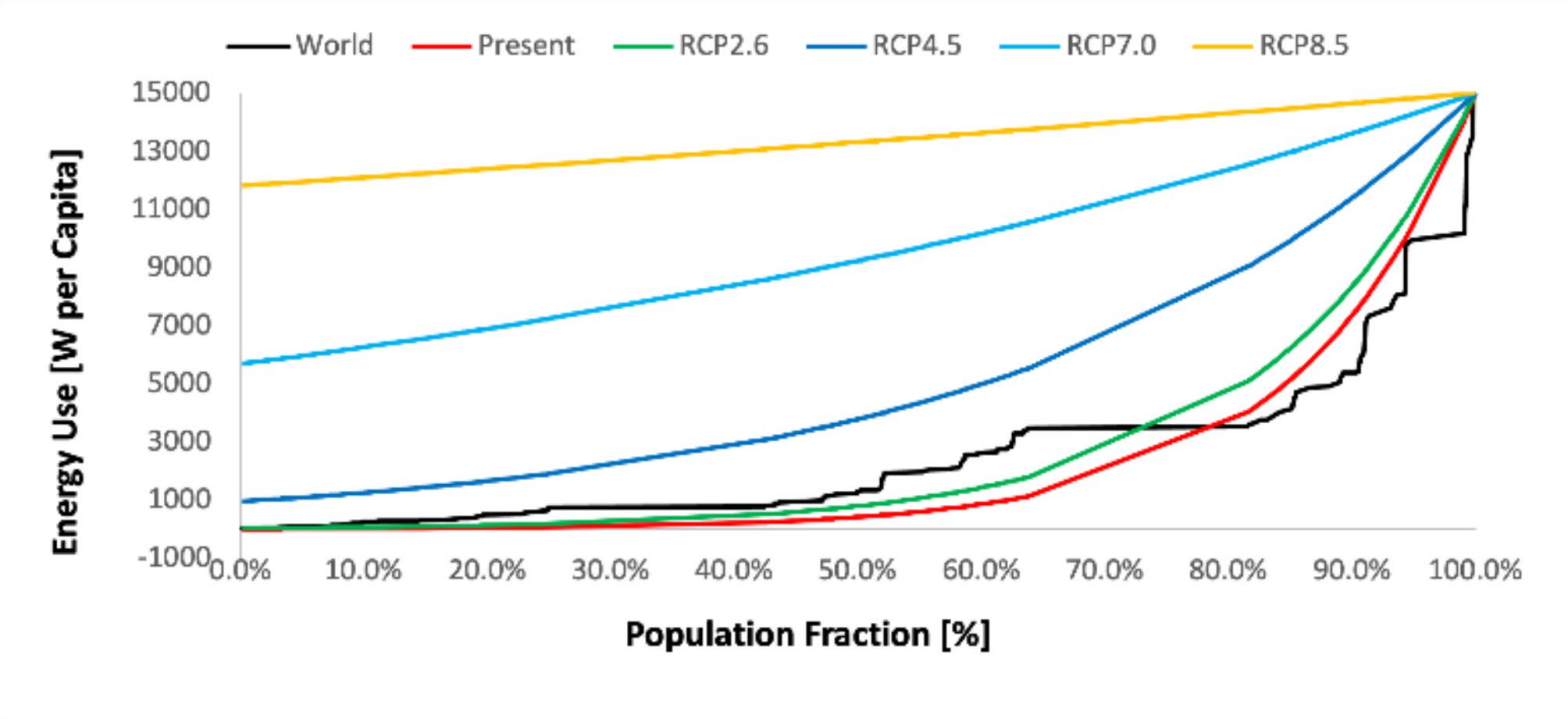
Global per capita energy use distribution. Present distribution (black curve) is based on country statistics from the CIA World Factbook. Colored curves are synthetic distributions following exponential curves averaging around the mean per capita energy consumptions of the respective RCP scenarios.

To express the socio-economic consequences of the different RCPs, the corresponding per capita energy consumptions were computed based on contemporary (8 billion) vs. projected future population (12 billion for 2100) (Table 2). Figure 3 shows the contemporary distribution of the per capita energy consumption based on country statistics from the CIA World Factbook. This distribution can be approximated by an exponential function, where the energy consumption of the poorest population $\:{p}_{0}=\:{p}_{min}$ is related to the richest $\:{p}_{1}={p}_{max}$ via an inequality ratio $\:R$ such that the per capita energy consumption over the entire population averages out to the mean per capita energy consumption $\:{p}_{mean}$ (Sect. 4.5). Assuming that the energy consumption of the richest population remains constant $\:{p}_{max}=\:\text{15,000}\left[W{\:cap}^{-1}\right]$ that is still ~50% higher than the current $\:\text{10,081}\left[W{\:cap}^{-1}\right]$ energy use in the United States, the distribution of the energy consumption can be computed for the average per capita energy consumption associated with the different RCPs (Fig. 3).

**Table 2:** Per capita energy consumptions in the present vs. at peak reaching the different RCPs considering present (8 billion) vs. future (12 billion) populations.

Scenario	Energy consumption [W capita^-1^]	
	8 billion people	12 billion people
Present	2625	1750
RCP 2.6	3079	2053
RCP 4.5	6240	4160
RCP 7.0	11,894	7989
RCP 8.5	16,533	11,022

**Table 3:** Socio-economic pathways.

SSP 1	Sustainability
SSP 2	Middle of the Road
SSP 3	Regional Rivalry
SSP 4	Inequality
SSP 5	Fossil-fueled development

The average per capita energy consumption (Table 2) along with the inequality between the richest and the poorest population is likely a better metric to express the economic consequences of the different representative concentration pathway trajectories than the “*shared socio-economic pathway*” (SSPs, yet another obfuscating acronym) scenarios (Table 3).

Based on Fig. 3, it is hard to justify the association of SSP 4 (inequality) with any of the higher RCPs. The disparity can only remain high if the highest energy consumers adopt insane energy consumption habits. Higher RCPs such as $\:{p}_{RCP4.5}=\:4160-6240\left[W{\:cap}^{-1}\right]$ (Table 2) have more room for even energy access without compromising the living standards in rich countries. The per capita ($\:{p}_{RCP2.6}=2053-3079\left[W{\:cap}^{-1}\right]$) energy consumption is less than a quarter of the current consumption in the US.

Unless the energy consumptions are met by non-fossil energy sources, the carbon dioxide emission targets represent a severe limit in alleviating energy poverty (Sect. 4.5). Global energy consumption in the upcoming decades has to increase by a minimum of 2–3 fold to end energy poverty and reach a more equitable future.

Scientific papers outlining energy transition relying entirely on renewable energy sources commonly assume that the present global energy consumption could be reduced. As an example, Jacobson et al.^16,17^ outlined a future where.

- 3.8 million 5 MW wind turbines.
- 49 thousand 300 MW concentrated solar farms,
- 40 thousand 300 MW photovoltaic solar farms,
- 1.7 billion 3 kW rooftop solar panels,
- 5.3 thousand 100 MW geothermal power plants,
- 270 new 1300 MW hydroelectric power plants,
- 730 thousand 0.75 MW wave devices, and.
- 490 thousand 1 MW tidal turbines.

power the World and provides 11.47[TW] power generation capacity. This would translate to 1462[W capita^− 1^] (about the per capita energy consumption in Ecuador) energy consumption at present (8 billion population) that would shrink to 956[W capita^− 1^] (below Namibia) when the population reaches 12 billion. Such low-energy access is likely impossible in colder regions, where heating and cooling alone will take up more energy^18^ therefore the renewable power generation infrastructure estimated by Jacobson et al.,^16,17^ has to be doubled or tripled at the minimum.

### Energy return on investment

Based on the total amount of energy used up when the different RCP levels are reached, both the exhausted portion and the remaining resources as a fraction of the total reserves can be computed (Table 1: *consumed energy at peak* and *remaining energy after peak*). These are valuable metrics to implement an adjustment to the number of years the remaining fossil fuel reserves and allow to factor in the declining energy return on investment (EROI)^19^ (Sect. 4.6). Traditionally, humanity succeeded in transitioning to poorer-quality resources by utilizing more energy^20^. This is possible to all resources except energy, where the energy return on the investment sets a firm limit, since energy costs exceeding return make energy extraction impossible^20^.

In the present analysis, a simple linear relationship was assumed between the energy cost of the energy production and the degree of depletion of the fossil fuel (Sect. 4.6). This approximation of the Energy Return on Investment (EROI)^19–22^ is likely to be overly optimistic, and chances are that energy costs will grow non-linearly as fossil resources are depleted. The presented linear approximation offers an estimate that can be carried out with simple analytic computations (Sect. 4.6).

The level of exhaustion $\:f=\frac{C-{\:C}_{base}}{{C}_{paleo}-{\:C}_{base}}=\frac{E}{{E}_{tot}}$ can be expressed from the atmospheric carbon dioxide concentration during paleo ($\:{C}_{paleo}=2000\left[ppm\right]$), pre-industrial $\:({\:C}_{base}=278\left[ppm\right])$, and present eras $\:({C}_{pres}=421\left[ppm\right])$ or from the ratio of the exhausted energy ($\:E$) and the total fossil fuel resources ($\:{E}_{tot}$). The energy cost of energy production can be expressed as $\:{P}_{cost}=k\:f\:P$, where $\:k$ is a cost coefficient and $\:P$ is the total energy produced.

There are considerable uncertainties in the EROI of the current fossil fuel extraction. For instance, a net energy analysis of the Bakken (North Dakota, USA) crude oil production using hydraulic fracturing found that the net energy return ranged between 13.3–52[MJ/MJ] (corresponding to 2–7.5% energy cost) with 29.3[MJ/MJ] median (approx. 3.4% energy cost)^23^. This cost does not include the energy cost of processing and distribution. One has to note that while the 2–7.5% energy cost with a 3.4% median might appear remarkably modest, the hydraulic fracturing process itself is already a sign of “*desperation*”. While opponents of hydraulic fracturing are mostly concerned about the environmental impacts, it is perhaps even more alarming that fossil fuel extraction reached the point when energy companies go after reserves that are in deep deposits that can be extracted only by injecting some fluid to open up the rock formations. This practice definitely will hit some limits when drilling deeper or injecting more fluid becomes infeasible.

In the absence of reliable data, we explored three scenarios with slow ($\:k=0.5$), medium ($\:k=1.0$) and rapid ($\:k=2.0$) EROI decline, leading to 4.15%, 8.3% and 16.3% energy cost at the contemporary $\:f=8.3\left[\%\right]$ exhaustion level. While the slow EROI decline when $\:k<1.0$ allows for the extraction of the entire remaining fossil fuel reserves, the rapid EROI decline leads to an additional constraint. At $\:{f}_{max}=\frac{1}{k}=\:50\%$ exhaustion level, the energy cost reaches 100%; therefore, energy extraction becomes impossible.

Under the slow EROI decline, the energy cost rises from 4.15 to 50% of the energy consumption. The net energy consumption continues to rise (but not exponentially) during the growth years (Table 1: *EROI decline—slow—net energy*). Under the rapid EROI decline scenario the net energy consumption peaks shortly after RCP 4.5 due to the rising energy costs (starting from 16.6% and exceeding 100% at RCP 8.5 and beyond, Table 1: *EROI decline—rapid—net energy*). When the cost of energy production exceeds the energy returned, the energy production becomes impossible.

The EROI is assumed to be included in the rising energy consumption during the exponential growth years. While this assumption is applicable in the slow EROI decline, the energy cost exceeding 100% at the peak of the exponential growth indicates that the accessible fossil fuel reserves were exhausted earlier therefore the exponential growth years need to be adjusted downward (Table 1—*EROI decline—rapid—constant years*). EROI was applied to the net energy consumption at the end of the exponential growth years, to find the remaining years with constant net energy consumption (Sect. 4.6) before the accessible fossil fuel reserves are exhausted (Table 1: *EROI decline—slow—constant years*,* EROI decline—medium—constant years* and *EROI decline—rapid—constant years*). One has to note that since RCP 8.5 and 2000ppm cannot be reached in the rapid EROI decline, therefore the Constant Years represent a negative adjustment between the total ($\:{f}_{total}=100\%$) and the accessible ($\:{f}_{max}=50\%$) fossil fuel reserves.

The years left from the present to the time when the accessible fossil fuel reserves are exhausted are computed from the sum of the growth and the constant years (Table 1: *EROI decline—slow—years left*, *EROI decline—medium—years left* and *EROI decline—rapid—years left*). It might seem odd that ”*years left*“ for RCP 7.5 and reaching 2000[ppm] (that both take 76 years when the accessible fossil fuel reserves are exhausted) exceed the *Years Left* for RCP 7.0 which is 71 years. This is due to the 7 constant years during which the net energy consumption is kept constant 23[TW] despite the rapidly declining EROI. We also have to note that net energy consumption returns to close to contemporary levels at 20[TW].

One has to mention that while some paleo records suggest that atmospheric carbon dioxide concentrations reached over 6000[ppm] (that is well above 2000[ppm]). If this were the case, the present-day EROI would imply a more rapid EROI decline. The approximately three-fold larger fossil fuel reserves in the light of energy cost of energy production between 4.3 and 16.6% would lead to $\:k\sim1.5,\:3.0\:or\:6.0$ corresponding to the slow, medium and rapid EROI decline scenarios. A $\:k=3.0$ scenario, when the energy cost of producing energy increases three times faster than the fraction of the exhausted fossil fuel reserves would render $\:1-{f}_{max}=\frac{2}{3}$ of the the remaining fossil fuels inaccessible (since the accessible fossil fuel reserves are$\:{f}_{max}=\frac{1}{k}\:$, Sect. 4.6).

These findings are not new. The “limits to growth”^24^ and Hubbert curve^25^ raised widely recognized awareness of the unsustainability of exponential growth on a finite planet that was repeatedly confirmed^22^. Expedited, transitioning away from fossil fuels is a must if humanity wants to avoid a rapid collapse^24^ very soon. Even those who are otherwise skeptical about the severity of climate change should be very much concerned about running out of fossil fuels soon.

### Carbon capture and sequestration

Energy consumption estimates presented in Sect. 2.3 are all based on assuming that fossil fuels will remain the primary source of energy, the energy consumption and exhausted energy estimates guide the magnitude of the energy transition and the feasibility of “*net-zero*” ambitions (a partial transition where carbon dioxide emissions are balanced by some form of removal from the Earth’s atmosphere). A recent paper^26^ estimating the energy storage requirement relying on solar or wind found the need for massive energy storage capacity in the order of 1/3rd or 1/4th of the annual energy consumption for 100% renewable penetration. In contrast, the vast majority of the installed energy storage facilities have only a few hours capacity. The large discrepancy is due to the currently installed storage solutions are only meant to mitigate diurnal or even higher frequency variabilities in renewable power generation, while much larger variations occur seasonally or inter-annually.

In the absence of storage capacity that can handle seasonal or inter-annual variations, the vast majority of the energy literature outlining the integration of renewable energy sources (primarily solar and wind) into the current mix of energy sources is limited to partial “*de-carbonation*” where 20–30% renewable contribution is considered high renewable “*penetration*”^26^. Some authors argued that integrating renewable energy sources beyond 50% into the energy system was impossible^27,28^. The inevitable continued reliance on fossil fuels has led to the widely accepted notion that carbon capture and sequestration (CCS) will be a critical technology in achieving “*net-zero*” carbon dioxide emissions^29,30^.

As a first approach to acting on climate change, biotic carbon capture sequestrations incentivized via various “*carbon trading*” schemes were envisioned (e.g. Kyoto Protocol treaty signed in 1997) assuming that emissions can be offset via sustainable land-use practices that promote ecosystems to uptake more carbon dioxide. Perhaps, the atmospheric carbon dioxide concentration variability (180–300[ppm]) during the Holocene (in the last 800,000 years)^11^ in contrast to the difference between paleo and pre-industrial atmospheric carbon dioxide concentration ($\:{C}_{paleo}-{C}_{base}=1722\left[ppm\right]$), approximating the fossil fuel reserves) could serve as a first-order estimation of the magnitude that biotic carbon capture and sequestration could contribute to the carbon dioxide removal from the atmosphere.

Industrial carbon capture as an alternative to biotic solutions has existed for decades. The typical applications for captured carbon dioxide include natural gas treatment and the production of hydrogen and other industrial chemicals^31^. Estimates for the increased fuel requirements of carbon capture at fossil fuel power plants vary (24–40% increase for pulverized coal power plants, 14–25% increase for integrated coal gasification combined cycle power plants, and 11–22% increase for natural gas power plants). In addition to capturing, the carbon dioxide will need to be transported to suitable geological locations. Only about 5,000 miles of carbon dioxide pipelines currently operate in the United States, primarily linking natural carbon dioxide sources to oil fields for enhanced oil recovery^31^. A much larger pipeline network would be needed for CCS to meet carbon dioxide reduction goals.

Beyond the energy demand of carbon capture and sequestration, one has to wonder where the captured carbon dioxide would be stored. Large-scale sequestration is largely envisioned by injecting carbon dioxides underground to porous sediment formation preferably to cavities left behind from fossil fuel extraction. Applying high school chemistry, the mass ratio of the emitted carbon dioxide vs. the burned fuel is 3.67 for coal, approximately 3.14 for liquid hydrocarbons, and 2.75 for natural gas (largely methane). Volumetrically, these ratios are 7.63 for coal, 3–3.9 for liquid hydrocarbons, and 1.55 for liquified natural gas (where the theoretical minimum energy cost of compressing the methane gas to liquid is 1.99% of its energy content). The theoretical minimum energy needed to compress carbon dioxide to liquid at the mean surface temperature of the Earth is 3.57% of its carbon content (Sect. 4.7).

Since the carbon dioxide resulting from burning fossil fuels has more mass and larger volume than the original fuel source, cavities left behind from fossil fuel extractions will not be able to hold the resulting carbon dioxide even in the liquid phase. Current estimates based on geological surveys of suitable sediment formation range between 8,000–55,000[Gt]^32^. In contrast, the carbon dioxide content that was removed from the atmosphere from the paleo times to the pre-industrial era $\:\left(2000\left[ppm\right]-278\left[ppm\right]\right)\times\:7.82\left[Gt\:pp{m}^{-1}\right]=\text{13,466}\left[Gt\right]$ exceeds the lower estimate of the sustainable sediment formations. One has to wonder about the ability of these porous sediment formations to hold large amounts of highly pressurized carbon dioxide without any leakage at temperatures likely much higher than the ambient air on the Earth’s surface (due to the geothermal gradient toward the Earth’s magma).

Besides the amount of carbon dioxide that might be sequestered, some authors attempted to estimate the rate at which the carbon dioxide sequestration could be carried out and found that the realistic rate is likely around 5–6[Gt yr^− 1^] with the possibility of reaching 16[Gt yr^− 1^] as a maximum^30^. This is significantly less than the up to 30[Gt yr^− 1^] estimate from IPCC^31^. To put these numbers into context, the carbon dioxide emission at the peak energy consumption was computed (Table 1: *Carbon capture and sequestration*) assuming that the fossil fuel consumption is stabilized at the peak energy consumption (so any further increase that will be necessary to eliminate energy poverty should come exclusively from non-fossil fuel sources). The resulting 27–31[Gt yr^− 1^] for continued present consumption or slight increase reaching RCP 2.6 suggests, that the IPCC estimate^31^ was based on more the desired capacity than feasibility.

Considering the excessive energy cost of capturing, transportation, and sequestration and the vast amount of carbon dioxide that needs to be stored, one has to wonder if carbon capture and sequestration is a viable technology (within the realm of true science) or if it is destined to fail and find its place next to pseudo sciences such as alchemy or astrology.

## Conclusions

The present paper attempted to estimate the plausible life span of the remaining fossil fuels by approximating the exhausted and remaining fossil fuel reserves from atmospheric carbon dioxide concentration as a fuel gauge. On this basis, the remaining fossil fuel reserves may last for a few hundred years at current total consumption (that includes the cost of energy production) but will be exhausted approximately within a Century if the total energy consumption continues to grow exponentially at the current 2.4% annual rate. Assuming a linear relationship between the cost of energy production (an inverse of the Energy Return on Investment, EROI) and assuming constant net energy consumption (with growing total energy consumption to account for the growing cost of energy production) the time left goes down to significantly (at present net energy consumption) or less than a Century (around 70 years) when the net energy consumption more than doubles, which is a must for the elimination of energy poverty. While none of the numbers computed in this paper are exact enough to take at face value, it is quite clear that none of the tested scenarios allow the continued use of fossil fuels for thousands of years and chances are that the remaining reserves will be exhausted within the life span of the next generation (the newly born grandchildren of the baby boom generation).

The paper attempts to bridge the carbon emission scenarios explored in recent IPCC reports that express “*representation concentration pathways*” in radiative forcing corresponding to the accumulated atmospheric carbon dioxide concentrations. Applying an inverted empirical relationship relating atmospheric carbon dioxide to radiative forcing, the “*representative concentration pathways*” were expressed as atmospheric carbon dioxide concentration. Assuming continued exponential growth in energy consumption at the current 2.4% annual rate, the time reaching the atmospheric carbon dioxide concentrations associated with the different “*representative concentration pathways*” were computed. Out of the four tested scenarios (RCP 2.6, RCP 4.5, RCP 7.0, and RCP 8.5), only RCP 8.5 will take more than 75 years to reach.

The paper also assessed the feasibility of the continued reliance on fossil fuels at some limited capacity and deploying carbon capture and sequestration technologies to achieve “*net-zero*” carbon dioxide emission. Considering the vast majority of the fossil fuels that are still in the ground, capturing, transporting and sequestering carbon dioxide when burned appears to be next to impossible even if suitable geological formation exists to store carbon dioxide. The energy cost of carbon capture and sequestration (likely in the range of 20–40% of the energy produced) will be prohibitively expensive as the cost of fossil fuel extraction and distribution increases.

To summarize these findings, this paper argues that transitioning to carbon-free energy sources is inevitable and urgent irrespective of our concerns about the anticipated impact of the elevated atmospheric carbon dioxide concentrations. This transition has to be “*true zero*” without compromise via carbon capture and sequestration or accounting gimmicks such as carbon trading. Revised focus on the energy transition to “*true zero*” energy sources will address climate change as a by-product, while the carbon dioxide emission goals could lead to “*solutions*” that ultimately expedite the exhaustion of the remaining fossil fuels without solving the core problems.

## Methods

The primary objective of the presented analysis is to express the relationships between energy consumption, atmospheric carbon dioxide concentration, and the exhaustion of fossil fuels in the form of back-of-the-envelope calculations that non-experts with a basic understanding of the underlying physics, chemistry, and math can reproduce on their own and are suitable for discussing these topics in freshman level undergraduate courses.

### Atmospheric carbon dioxide content

In the context of carbon dioxide emissions or concentration, it appears to be customary to use carbon dioxide and carbon interchangeably. Since this leads to confusion about when carbon dioxide concentration is related to the energy content of the carbon burned, this paper makes a deliberate effort to distinguish them.

The atmospheric carbon dioxide concentration can be turned into the total amount of carbon dioxide by considering the total mass of the atmosphere ($\:{m}_{atm}\:=\:5.15\times\:{10}^{6}\left[Gt\right]$)^33^. Neglecting the change in the atmospheric mass when some of its gas mixture is partially replaced by volume with higher density carbon dioxide, the mass of the carbon dioxide in the atmosphere can be expressed as a function of its concentration ($\:C$ in ppm) by considering the average molecular mass of the atmospheric gas composition ($\:{M}_{atm}=\:28.96$) and the carbon dioxide ($\:{M}_{{CO}_{2}}={M}_{C}\:+\:2{\:M}_{O}=\:44$) as:

1 $$\:{m}_{atm,{CO}_{2}}\:=C\times\:{10}^{-6}\frac{{M}_{{CO}_{2}}}{{M}_{atm}}{m}_{atm}=C\:7.82\left[Gt{\:ppm}^{-1}\right]$$

Considering that the atmospheric carbon dioxide concentration increased $\:{C}_{pres}-{C}_{base}=143\left[ppm\right]$ since the beginning of the Industrial Revolution, the added carbon dioxide was $\:{\varDelta\:m}_{{pres,CO}_{2}}\:=1119\left[Gt\right]$.

The carbon dioxide emissions from burning coal that produces a unit amount of energy (often referred to—somewhat inaccurately—as “*carbon intensity*”) is equal to:

2 $$\:{e}_{coal}=\frac{1}{{h}_{coal}}\frac{{M}_{{CO}_{2}}}{{M}_{C}}=\frac{1}{32.8\left[kJ{{\:g}_{C}}^{-1}\right]}\frac{44\left[g{\:mol}^{-1}\right]}{12\left[g{\:mol}^{-1}\right]}=111.8\left[{g}_{{CO}_{2}\:}{\:MJ}^{-1}\right]$$

where $\:{h}_{coal}=32.8\left[kJ{{\:g}_{coal}}^{-1}\right]=1.040\left[TWyr{\:Gt}^{-1}\right]$ is the energy released from burning a unit mass of coal.

Similarly, the carbon dioxide emission from burning hydro-carbonates ($\:{C}_{x}{H}_{2x+2}$) is:

3 $$\:{e}_{{C}_{x}{H}_{2x+2}}=\frac{1}{{h}_{{C}_{x}{H}_{2x+2}}}\frac{{x\:M}_{{CO}_{2}}}{{M}_{{C}_{x}{H}_{2x+2}}}$$

Table 4 shows the contribution of fossil fuels by the three major types (coal, oil, and gas) in the global energy mix as of 2020. We introduce *carbon portion* ($\:{I}_{fossil}$) as a percentage of the energy arising from burning carbon in energy production. The *carbon portion* of the energy mix can be computed by considering the share of different fuel types (coal, oil, and gas) and their respective *carbon/CO*_*2*_ *intensity* (Table 4) yielding $\:{I}_{fossil}=67.4\%$ for the current fossil fuel mix.

**Table 4:** Computation of the carbon intensity of the fossil fuel energy mix.

	Global energy share (%)	Fossil fuel share (%)	Energy content $\:\left[{kJ\:g\:}^{-1}\right]$	CO_2_ intensity$\:{\left[{{\:g}_{{CO}_{2}}MJ}^{-1}\right]}_{\:}$	Carbon portion (%)	Carbon share (%)
Coal	27.2	32.3	32.8	111.8	100.0	32.3
Oil	32.2	38.3	48.0	64.3	57.5	22.0
Gas	24.7	29.4	55.6	49.5	44.3	13.0
**Total**	**84.1**	**100.0**		**75.3**		**67.4**

The product of the energy contribution of fossil fuels ($\:{F}_{fossil}=84\%$) and the *carbon portion* of the fossil fuels mix ($\:{I}_{fossil}=67.4\%$), $\:{I}_{C}={I}_{fossil}{F}_{fossil}=56.6\%$) can serve as the *carbon portion* of the entire energy mix.

According to the Global Carbon Budget, only 44% of the total carbon dioxide emission between 1850 and 2019 found its way to the atmosphere, so the total emission was $\:{\varDelta\:m}_{{tot,CO}_{2}}=\frac{{\varDelta\:m}_{{pres,CO}_{2}}}{44\%}=\frac{1119\:\left[Gt\right]}{44\%}=2543\:\left[Gt\right]$. One-third of the total carbon dioxide emission was due to land-use change, and the rest $\:{\varDelta\:m}_{{fossil,CO}_{2}}=1695\left[Gt\right]$ was from burning fossil fuels ($\:{\varDelta\:m}_{fossil,C}=\frac{{M}_{C}}{{M}_{{CO}_{2}}}\varDelta\:{m}_{{fossil,CO}_{2}}=462\left[Gt\right]$, carbon) was from burning fossil fuels, representing $\:{E}_{fossil,C}={{h}_{C}\varDelta\:m}_{fossil,C}=481\left[TWyr\right]$ energy. Applying the *carbon portion* ($\:{I}_{C}=56.6\%$) of the energy mix, the exhausted energy up to the present can be estimated based on the carbon budget:

4 $$\:{E}_{pres,CB}=\frac{{E}_{fossil,C}}{{I}_{C}}=849\left[TWyr\right]$$

### Exponential energy consumption trajectories

This paper clearly distinguishes energy consumption as a rate of energy flow (the unit of [k, M, G, T]**W** that is called power in physics) from the total accumulated/exhausted energy used up to a given time. It has to be noted that the energy literature adopted a somewhat confusing habit of expressing both annual, monthly, daily, etc. consumption and total energy in energy units such as [k, M, G, T, P]**Whr**, **BTU**s, etc. While energy units are appropriate for discussing energy reserves or storage capacities, the use of power units would be clearer when used in the context of consumption^26^. Energy consumption over unit time expressed in energy units carries a hidden yr^− 1^ dimension. Once the hidden dimension is added to the energy units [k, M, G, T, P]**Whr yr**^**− 1**^, the resulting unit becomes power times a unit-less **hr yr**^**− 1**^ multiplication coefficient. The authors of the present paper are not alone in this assertion, and Sir David MacKay (a physicist and former science advisor to the UK Department of Energy and Climate Change) came to the same conclusion^8^.

At the core of our analysis is the approximation of the temporal evolution of global energy consumption as an exponential function arising from a steady annual growth rate ($\:r=2.4\%$)^1^. The resulting exponential relationship between time and rate of energy consumption ($\:P$) is:

5 $$\:P\:={\:P}_{0}{\left(1+r\right)}^{t}={P}_{0}{e}^{ln\left(1+r\right)t}$$

where $\:{P}_{0}$ is the initial energy consumption $\:{t}_{0}=0$.

The amount of energy ever consumed going back to $\:{t}_{begin}=-\infty\:$ can be expressed by integrating Eq. (8):

6 $$\:E\:={\:P}_{0}\underset{-\infty\:}{\overset{t}{\int\:}}{\left(1+r\right)}^{t}dt=\frac{{P}_{0}}{ln\left(1+r\right)}\left[{\left(1+r\right)}^{t}-{\left(1+r\right)}^{-\infty\:}\right]=\frac{{P}_{0}{\left(1+r\right)}^{t}}{{ln}\left(1+r\right)}=\frac{P}{\text{ln}\left(1+r\right)}$$

Since $\:{E}_{0}=\frac{{P}_{0}}{\text{ln}\left(1+r\right)}$, this equation can be expressed as $\:E={E}_{0}{\left(1+r\right)}^{t}$ showing that the integral of an exponential function increases at the same rate as the exponential function itself. Applying this equation to the present energy consumption, $\:{P}_{pres}=21\left[TW\right]$ leads to $\:{E}_{pres}=885\left[TWyr\right]$ exhausted energy up to the present.

From $\:P={P}_{0}{\left(1+r\right)}^{t}$ or $\:E={E}_{0}{\left(1+r\right)}^{t}$ the elapsed time while the power consumption/exhausted energy increases from an initial value $\:{P}_{0}$ or $\:{E}_{0}$ to a new level ($\:P$ and $\:E$) can be expressed as:

7 $$\:t=\frac{\text{ln}\left(\frac{E}{{E}_{0}}\right)}{\text{ln}\left(1+r\right)}=\frac{\text{ln}\left(\frac{P}{{P}_{0}}\right)}{\text{ln}\left(1+r\right)}$$

### Fossil fuels from carbon dioxide

Assuming that exhausted energy up to the present is proportional to the atmospheric carbon dioxide concentration increment ($\:{C}_{pres}-{C}_{base}$), and the total fossilized reserves are proportional to the atmospheric concentration decline from the paleo ($\:{C}_{paleo}$) to the pre-industrial ($\:{C}_{base}$) era the total energy in the fossilized reserves can be expressed as:

8 $$\:{E}_{tot}={\frac{{C}_{paleo}-{\:C}_{base}}{{C}_{pres}-{\:C}_{base}}E}_{pres}=\text{10,663}\left[TWyr\right]$$

This equation can be rearranged to:

9 $$\:{E}_{pres}={\frac{{C}_{pres}-{\:C}_{base}}{{C}_{paleo}-{\:C}_{base}}E}_{tot}$$

where $\:f=\frac{{E}_{pres}}{{E}_{tot}}=\frac{{C}_{pres}-{\:C}_{base}}{{C}_{paleo}-{\:C}_{base}}=8.3\%$ can be viewed as the exhausted portion of the fossil fuel reserves.

### Radiative forcing from carbon dioxide concentration

The solar radiation reaching the troposphere of the earth $\:{F}_{Sun}=1360\left[W{\:m}^{-2}\right]$ is partially reflected and the rest is absorbed $\:{F}_{abs}=\left(1-a\right){F}_{Sun}\frac{{A}_{circle}}{{A}_{sphere}}=238\left[W{\:m}^{-2}\right]$ (where $\:a=0.3$ is the albedo of the Earth) on the cross-sectional area of the Earth ($\:{A}_{circle}={R}^{2}\pi\:$) that is equal to the projected area facing the Sun. To balance out the absorbed radiation, the Earth has to emit the same radiation over its surface area ($\:{A}_{sphere}={4R}^{2}\pi\:$) following the Stefan-Boltzmann law so its planetary surface temperature can be expressed as:

10 $$\:{A}_{sphere}{F}_{abs}={A}_{sphere}\sigma\:\left({T}_{planet}^{4}-{T}_{space}^{4}\right)\to\:{T}_{planet}=\sqrt[4]{\frac{{F}_{abs}}{\sigma\:}}=254.5\left[K\right]$$

where the $\:{T}_{space}\approx\:0\left[K\right]$ and $\:\sigma\:=5.67\times\:{10}^{-8}\left[W\:{m}^{-2}{K}^{-4}\right]$ is the Stefan-Boltzmann constant. The planetary surface temperature $T_{{planet}} = - 18.61\left[ {^\circ {\text{C}}} \right]$ is $\:\varDelta\:{T}_{const}={T}_{Earth}-{T}_{planet}=33.61\left[ {^\circ {\text{C}}} \right]$ colder than the average surface temperature of the Earth ($\:{T}_{Earth}=15\left[ {^\circ {\text{C}}} \right]$) which is attributed to the effect of greenhouse gases. The adjusted planetary temperature resulting from the radiative forcing increment ($\:\varDelta\:F$ that the representative concentration pathways represent) can be computed from the Stefan-Boltzmann law:

11 $$\:{T}_{planet}^{{\prime\:}}=\sqrt[4]{\frac{{F}_{abs}+\varDelta\:F}{\sigma\:}}$$

so, the temperature increment ($\:{\varDelta\:T}_{{CO}_{2}}$) as a function of the radiative forcing can be expressed as:

12 $$\:{\varDelta\:T}_{{CO}_{2}}={T}_{planet}^{{\prime\:}}-{T}_{planet}=\sqrt[4]{\frac{{F}_{abs}+\varDelta\:F}{\sigma\:}}-\sqrt[4]{\frac{{F}_{abs}}{\sigma\:}}$$

The radiative forcing of carbon dioxide as a greenhouse gas can be expressed as a function of its concentration as^15^:

13 $$\:\varDelta\:F=5.32\:\text{ln}\left(\frac{C}{{C}_{0}}\right)+0.39{\left[\text{ln}\left(\frac{C}{{C}_{0}}\right)\right]}^{2}$$

For known changes of the radiative forcing (that the Representation Concentration Pathways express) the corresponding carbon concentration becomes:

14 $$\:\text{ln}\left(\frac{C}{{C}_{0}}\right)=\frac{-5.32\pm\:\sqrt{{5.32}^{2}+4\times\:0.39\varDelta\:F}}{2\times\:0.39}\to\:C={\:C}_{0}{e}^{\frac{-5.32\pm\:\sqrt{28.3+1.56\varDelta\:F}}{0.78}}\:$$

### Per capita energy consumption as socio-economic metric

The distribution of the per capita energy consumption ($\:p$) from the lowest to the highest (plotted from country statistics provided by the CIA World Factbook, Fig. 3) appears to follow an exponential relationship ($\:p={p}_{0}{R}^{x}$) that can be characterized by the poorest $\:{\:p}_{o}={\:p}_{min}$and the richest $\:{\:p}_{max}$, where the ratio of the richest over the poorest $\:R={\:p}_{max}/{\:p}_{min}$ could serve as a measure of inequality. Expressing the lowest energy consumption from this ratio $\:{\:p}_{min}={p}_{0}={\:p}_{max}/R$, the global mean energy and the ratio of the mean energy and the maximum ($\:{p}_{mean}/{p}_{max}$) per capita energy consumption can be expressed as:

15 $$\:{p}_{mean}\:=\frac{{\:p}_{max}}{R}\underset{0}{\overset{1}{\int\:}}{R}^{x}dx=\frac{{p}_{max}}{R}{\left[\frac{{R}^{x}}{\text{ln}\left(R\right)}\right]}_{0}^{1}=\frac{{p}_{max}}{R\:\text{ln}\left(R\right)}\left(R-1\right)\to\:\frac{{p}_{mean}}{{p}_{max}}=\frac{R-1}{R\:\text{ln}\left(R\right)}$$

This equation does not have an analytic solution, but equation solvers like WolframAlpha can solve it numerically to find the inequality ratio for any given $\:{p}_{mean}/{p}_{max}$ ratios.

When inequality is $\:R=1$, the equation leads to uniform energy access $\:{{p}_{min}{=p}_{mean}=p}_{max}$:

16 $$\:{p}_{mean}=\underset{R\to\:1}{\text{lim}}\:\:\frac{{p}_{max}}{R}\frac{R-1}{\text{ln}\left(R\right)}={p}_{max}$$

### Energy return on investment

Assuming a linear relationship between the cost of energy ($\:{P}_{cost}$) and the fraction of exhausted fossil fuel reserves ($\:f=\frac{E}{{E}_{tot}}$), the energy cost of extracting fossil fuel can be expressed as:

17 $$\:{P}_{cost}=k\frac{E}{{E}_{tot}}P$$

where $\:k$ is a constant coefficient. The net energy after deducting the cost of energy becomes:

18 $$\:{P}_{net}=P\left(1-k\frac{E}{{E}_{tot}}\right)$$

When the coefficient is $\:k\le\:1$ all the total fossil fuel reserves ($\:{E}_{tot}$) are available. When $\:k>\:1$ the accessible reserves are limited to $\:k\frac{{E}_{max}}{{E}_{tot}}=1$ therefore:

19 $$\:{E}_{max}=\left\{\begin{array}{ccc}{E}_{tot}&\:\to\:&\:k\le\:1\\\:\frac{{E}_{tot}}{k}&\:\to\:&\:k>1\end{array}\right.$$

Assuming the energy costs are included in the growth of the energy consumption ($\:P$) during the exponential growth phase, a steadily growing portion of the energy consumption goes toward extracting energy:

20 $$\:{P}_{net}=P\left(1-k\frac{E}{{E}_{tot}}\right)=E\:{ln}\left(1+\:r\right)\left(1-k\frac{E}{{E}_{tot}}\right)=E\:\text{ln}\left(1+r\right)-k\frac{{E}^{2}}{{E}_{tot}}\text{ln}\left(1+r\right)$$

that will peak at $\:1-\frac{2k}{{E}_{tot}}{E}_{peak}=0{\to\:E}_{peak}=\frac{{E}_{tot}}{2k}$.

For stabilized energy consumption starting at the end of the exponential growth phase ($\:{P}_{0}={E}_{0}\text{ln}\left(1+r\right)$), the constant net energy consumption can be expressed as: $\:{P}_{net}={P}_{0}\left(1-k\frac{{E}_{0}}{{E}_{tot}}\right)$.

Since $\:P={\frac{dE}{dt}={\:P}_{net}+P}_{cost}$ the change in exhausted energy over time leads to a differential equation:

21 $$\:\begin{array}{ccc}\frac{dE}{dt}&\:=&\:{P}_{net}+k\frac{E}{{E}_{tot}}\frac{dE}{dt}\\\:\int\:\left(1-k\frac{E}{{E}_{tot}}\right)dE&\:=&\:{P}_{net}\int\:dt\\\:E-\frac{k}{2}\frac{{E}^{2}}{{E}_{tot}}&\:=&\:{P}_{net}t+c\end{array}$$

Solving constant $\:c$ for $\:{t}_{0}=0$, when the exhausted energy ($\:{E}_{0}$) yields:

22 $$\:c={E}_{0}-\frac{k}{2}\frac{{E}_{0}^{2}}{{E}_{tot}}$$

Once, the constant $\:c$ is known the time $\:t$ to reach exhausted energy ($\:E$) can be computed as:

23 $$\:t\:=\frac{E\:-{E}_{0}-k\:\frac{{E}^{2}-{E}_{0}^{2}}{{2\:E}_{tot}}}{{P}_{net}}=\frac{\left(E-{E}_{0}\right)\left(1-k\frac{E+{E}_{0}}{{2E}_{tot}}\right)}{{P}_{net}}$$

Applying this equation for the accessible fossil fuel reserves ($\:{E}_{max}$) the remaining time ($\:{t}_{const}$) for constant net energy consumption can be computed. When the exhausted energy at the end of the growth phase exceeds the accessible fuel reserves $\:{{E}_{0}>E}_{max}$ the time reaching $\:{E}_{0}$ needs to be adjusted via a negative $\:{t}_{const}$ using Eq. 5, so:

24 $$\:{t}_{const}=\left\{\begin{array}{ccc}\frac{\left(E-{E}_{0}\right)\left(1-k\frac{E+{E}_{0}}{{2E}_{tot}}\right)}{{P}_{net}}&\:\to\:&\:{E}_{0}<{E}_{max}\\\:-\frac{\text{ln}\left(\frac{{E}_{tot}}{{E}_{max}}\right)}{\text{ln}\left(1+r\right)}&\:\to\:&\:{E}_{0}\ge\:{E}_{max}\end{array}\right.$$

Equation 24 can be rearranged to express exhausted energy as a function of time that leads to a quadratic equation:

25 $$\:t{\:P}_{net}{E}_{tot}=E{E}_{tot}-{E}_{0}{E}_{tot}-\frac{k}{2}{{E}^{2}+\frac{k}{2}E}_{0}^{2}$$

$$\:{\frac{k}{2}E}^{2}-E{\:E}_{tot}+{E}_{0}{E}_{tot}-\frac{k}{2}{E}_{0}^{2}+{P}_{net}{E}_{tot}t=0$$

26 $$\:E=\frac{{E}_{tot}\pm\:\sqrt{{E}_{tot}^{2}-2k\left({E}_{0}{E}_{tot}-\frac{k}{2}{E}_{0}^{2}+{P}_{net}{E}_{tot}t\right)}}{k}$$

### Carbon capture and sequestration

The fundamental challenge of carbon capture and sequestration is that any form of fossil fuels, when burned, leads to more mass as carbon dioxide than the fuel itself:

$$\:\begin{array}{cccccc}Coal&\:\frac{{M}_{{CO}_{2}}}{{M}_{C}}&\:=&\:\frac{44\left[g{\:mol}^{-1}\right]}{12\left[g{\:mol}^{-1}\right]}&\:=&\:3.67\\\:Methane&\:\frac{{M}_{{CO}_{2}}}{{M}_{{CH}_{4}}}&\:=&\:\frac{44\left[g{\:mol}^{-1}\right]}{16\left[g{\:mol}^{-1}\right]}&\:=&\:2.75\\\:Oil/Gasoline&\:\frac{x\:{M}_{{CO}_{2}}}{{M}_{{C}_{x}{H}_{2(x+1)}}}&\:=&\:\frac{x\:44\left[g{\:mol}^{-1}\right]}{12x+2\left(x+1\right)}&\:\cong\:&\:3.14\end{array}\:$$

In addition to the higher mass, the density of liquid carbon dioxide ($\:{\rho\:}_{liq,{\:CO}_{2}}=750.0\left[g{l}^{-1}\right]$) is less than any solid and most of the liquid fossil fuels, leading to volumetric increases: $R_{{V,Coal}} = \frac{{M_{{CO_{2} }} }}{{\rho _{{CO_{2} }} }} \div \frac{{M_{C} }}{{\rho _{C} }} = ~7.63$ for coal ($\:{\rho\:}_{C}=1560\left[g\:{l}^{-1}\right]$), $R_{{V,C_{x} H_{{2\left( {x + 1} \right)}} }} = \frac{{M_{{CO_{2} }} }}{{\rho _{{CO_{2} }} }} \div \frac{{M_{{C_{x} H_{{2\left( {x + 1} \right)}} }} }}{{\rho _{{C_{x} H_{{2\left( {x + 1} \right)}} }} }} = ~3 - 3.9$ for hydrocarbons ($\:{\rho\:}_{{C}_{x}{H}_{2\left(x+1\right)}}=718-940\left[g\:{l}^{-1}\right]$) and $R_{{V,CH_{4} }} = \frac{{M_{{CO_{2} }} }}{{\rho _{{CO_{2} }} }} \div \frac{{M_{{CH_{4} }} }}{{\rho _{{CH_{4} }} }} = ~1.55$ for natural gas (methane, $\:{\rho\:}_{{lig,CH}_{4}}=422.8\left[g{\:l}^{-1}\right]$).

The work associated with the expansion and compression of gases assuming constant temperature can be expressed by integrating the pressure ($\:p$) as the volume ($\:V$) changes:

27 $$\:W=\underset{{V}_{1}}{\overset{{V}_{2}}{\int\:}}pdV=n\:R\:T\:\text{ln}\left(\frac{{V}_{2}}{{V}_{1}}\right)$$

where the pressure can be expressed via the ideal gas law $\:p\:V=n\:R\:T$ with constant gas temperature ($\:\:T$), universal gas constant ($\:R$), and the number of molecules ($\:n=\frac{m}{M}$) computed from the ratio of the mass ($\:m$) and molecular mass ($\:M$) of the gas. The work for unit mass can be computed by dividing by the mass of the gas and replacing the volume ratio by the density ($\:\rho\:$) ratio of the gas before and after the volumetric change that yields:

28 $$\:w\:=\:\frac{W}{m}=\frac{\:R\:T\:}{M}\text{ln}\left(\frac{{\rho\:}_{1}}{{\rho\:}_{2}}\right)$$

Applying this equation to carbon dioxide in gaseous ($\:{\rho\:}_{gas,{\:CO}_{2}}=\:2\:\left[g{\:l}^{-1}\right]$) and liquid ($\:{\rho\:}_{liq,{\:CO}_{2}}=750\:\left[g{\:l}^{-1}\right]$) form at room temperature the energy needed for its compression can be computed. In contrast, from the chemical energy of burning carbon $\:{h}_{C}=\:32.8\left[kJ{\:g}^{-1}\right]$ the energy cost of liquifying carbon dioxide $\left( {\epsilon _{{liq}} = \frac{w}{{q_{{CO_{2} }} }}} \right)$ can be computed.

$$\begin{array}{*{20}c} {w_{{CO_{2} }} } & = & {\frac{{8.3145\left[ {J~mol^{{ - 1}} K^{{ - 1}} } \right]\left( {273.15 + ~15} \right)\left[ K \right]}}{{\left( {12 + 2 \times 16} \right)\left[ {g~mol^{{ - 1}} } \right]}}ln\left( {\frac{{750\left[ {kg~m^{{ - 3}} } \right]}}{{2\left[ {kg~m^{{ - 3}} } \right]}}} \right) = 322\left[ {J~g^{{ - 1}} } \right] = 0.322\left[ {G~J~t^{{ - 1}} } \right]} \\ {q_{{CO_{2} }} } & = & {\frac{{M_{C} }}{{M_{{CO_{2} }} }}h_{C} = \frac{{~12\left[ {g~mol^{{ - 1}} } \right]}}{{44\left[ {g~mol^{{ - 1}} } \right]}}32.8\left[ {kJ~g^{{ - 1}} } \right] = 9.03\left[ {G~J~t^{{ - 1}} } \right]} \\ {\epsilon _{{liq}} } & = & {\frac{w}{{Q_{{CO_{2} }} }} = \frac{{0.322\left[ {GJ~t^{{ - 1}} } \right]}}{{9.03\left[ {GJ~t^{{ - 1}} } \right]}} = 3.57\left[ {\text{\% }} \right]} \\ \end{array}$$

This is the theoretical minimum while the true energy cost of liquefying carbon dioxidearb is probably much higher.

The same computation for methane ($\:{\rho\:}_{gas,{\:CH}_{4}}=\:0.657\:\left[g{\:l}^{-1}\right]$ And $\:{\rho\:}_{liq,{\:CH}_{4}}=422.8\left[g{\:l}^{-1}\right]$, $\:{h}_{{CH}_{4}}=55.6\:\left[kJ{\:g}^{-1}\right]$) will yield:

$$\begin{array}{*{20}c} {w_{{CH_{4} }} } & = & {\frac{{8.3145\left[ {J~mol^{{ - 1}} K^{{ - 1}} } \right]\left( {273.15 + ~15} \right)\left[ K \right]}}{{\left( {12 + 4} \right)\left[ {g~mol^{{ - 1}} } \right]}}ln\left( {\frac{{422.8\left[ {kg~m^{{ - 3}} } \right]}}{{0.647\left[ {kg~m^{{ - 3}} } \right]}}} \right) = 1.109\left[ {G~J~t^{{ - 1}} } \right]} \\ {\epsilon _{{liq}} } & = & {\frac{w}{{h_{{CH_{4} }} }} = \frac{{1.109\left[ {GJ~t^{{ - 1}} } \right]}}{{55.06\left[ {GJ~t^{{ - 1}} } \right]}} = 1.99\left[ {\text{\% }} \right]} \\ \end{array}$$

## Electronic supplementary material

Below is the link to the electronic supplementary material.

Supplementary Material 1

## Data Availability

All data generated or analyzed during this study are included in this published article and its supplementary information files.

## Glossary

$\:{C}_{base}$: Atmospheric CO_2_ concentration before the industrial era, ppm
$\:{C}_{paleo}$: Atmospheric CO_2_ concentration before the fossil fuel accumulation started, ppm
$\:{C}_{pres}$: Atmospheric CO_2_ concentration in the present, ppm
$\:{C}_{RCPx.x}$: Atmospheric CO_2_ concentration reaching RCP scenarios, ppm
$\:{\varDelta\:C}_{pres}$: Atmospheric CO_2_ concentration increment from pre-industrial to present era, ppm
$\:{\varDelta\:C}_{res}$: Atmospheric CO_2_ concentration increment from present day to paleo level, ppm
$\:E$: Exhausted energy, TWyr
$\:{E}_{fossil,C}$: Exhausted energy up to present from burning carbon, TWyr
$\:{E}_{fossil,CB}$: Exhausted energy up to present from carbon budget, TWyr
$\:{E}_{peak}$: Exhausted energy at the end of exponential growth in energy consumption, TWyr
$\:{E}_{pres}$: Exhausted energy up to present from exponentially growing consumption, TWyr
$\:{E}_{pres,CB}$: Energy equivalent of the carbon increment in the atmosphere, TWyr
$\:{E}_{res,C}$: Energy content of the carbon from the added CO_2_ when paleo level is reached, TWyr
$\:{E}_{2000ppm}$: Exhausted energy when CO_2_ concentration returns to paleo level TWyr
$\:{E}_{tot}$: Total exhausted energy, TWyr
$\:f$: Fraction of the exhausted fossil fuel reserves, %
$\:{f}_{max}$: Maximum fraction of the fossil fuel reserves that can be explored, %
$\:{F}_{fossil}$: Fossil fuel contribution to the energy mix, %
$\:{\varDelta\:F}_{sol}$: Radiative forcing increase since the paleo era, W m^−2^
$\:{I}_{C}$: Carbon intensity of the energy mix, %
$\:{I}_{fossil}$: Carbon intensity of the fossil fuel mix, %
$\:k$: Coefficient relating the exhausted fossil reserves to the cost of energy
$\:{M}_{C}$: Molecular mass of carbon, g mol^−1^.
$\:{M}_{{CO}_{2}}$: Molecular mass of CO_2_, g mol^−1^
$\:{M}_{{CH}_{4}}$: Molecular mass of methane, g mol^−1^
$\:{M}_{{{C}_{x}H}_{2\left(x+1\right)}}$: Molecular mass of hydrocarbons, g mol^−1^
$\:{M}_{O}$: Molecular mass of oxygen, g mol^−1^
$\:{m}_{atm}$: Mass of the atmosphere, Gt
$\:{\varDelta\:m}_{fossil,C}$: Mass of the released carbon from burning fossil fuels up to present, Gt
$\:{\varDelta\:m}_{fossil{CO}_{2}}$: Mass of the released CO_2_ from burning fossil fuels up to present, Gt
$\:{\varDelta\:m}_{paleo,C}$: Mass decline of atmospheric carbon from paleo periods to present, Gt
$\:{\varDelta\:m}_{paleo,{CO}_{2}\:}$: Mass decline of atmospheric CO_2_ from paleo periods to present, Gt
$\:{\varDelta\:m}_{pres,C\:}$: Mass increment of the carbon from atmospheric CO_2_, Gt
$\:{\varDelta\:m}_{pres{,CO}_{2}\:}$: Mass increment of atmospheric CO_2_ since the industrial revolution, Gt
$\:{\varDelta\:m}_{res,C\:}$: Mass increment of the carbon from CO_2_ when concentration is at paleo level, Gt
$\:{\varDelta\:m}_{res,{CO}_{2}\:}$: Mass increment of the atmospheric CO_2_ when concentration is at paleo level, Gt
$\:{\varDelta\:m}_{{tot,CO}_{2}}$: Total amount of CO_2_ emission (fossil fuels and land use change combined), Gt
$\:P$: Global energy consumption, TW
$\:{P}_{cost}$: Energy cost of producing energy, TW
$\:{P}_{net}$: Net energy consumption, TW
$\:{P}_{peak}$: Global energy consumption at the peak of the exponential growth period, TW
$\:{P}_{pres}$: Global energy consumption at present, TW
$\:p$: Per capita energy consumption, W cap^−1^
$\:{p}_{max}$: Per capita energy consumption of the richest population, W cap^−1^
$\:{p}_{mean}$: Global mean per capita energy consumption, W cap^−1^
$\:{p}_{min}$: Per capita energy consumption of the poorest population, W cap^−1^
$\:{p}_{RCPx.x}$: Per capita energy consumption associated with RCP, W cap^−1^
$\:r$: Growth rate of the energy consumption
$\:R$: Inequality ratio of the richest and poorest by per capita energy consumption
$\:{\rho\:}_{gas,{\:CO}_{2}}$: Density of CO_2_ at atmospheric pressure as gas, g l^−1^
$\:{\rho\:}_{gas,{\:CH}_{4}}$: Density of methane at atmospheric pressure as gas, g l^−1^
$\:{\rho\:}_{liq,{\:CO}_{2}}$: Density of liquid CO_2_, g l^−1^
$\:{\rho\:}_{liq,{\:CH}_{4}}$: Density of liquid methane, g l^−1^
$\:{\rho\:}_{{C}_{x}{H}_{2\left(x+1\right)}}$: Density of hydrocarbons, g l^−1^
